# Targets and outcomes of psychotherapies for mental disorders

**DOI:** 10.1192/j.eurpsy.2021.35

**Published:** 2021-08-13

**Authors:** P. Cuijpers

**Affiliations:** Department of Clinical, Neuro and Developmental Psychology, Vrije Universiteit Amsterdam, Amsterdam, Netherlands

## Abstract

Because the causal pathways to mental disorders are largely unknown, it is also difficult to decide what the targets and outcomes of psychotherapies should be. In this presentation I will give an overview of the main types of targets and outcomes of therapies, as well as a brief overview of some of the main results of research on these types. The most important outcomes are symptom reduction, personal targets and outcomes from the patient’s perspective, improvement of quality of life, intermediate outcomes depending on the theoretical framework of the therapist, negative outcomes, and economic outcomes. By far the most research has focused on symptom reduction. In this presentation I will focus on psychotehrapies in general, but will illustrate the findings with research on psychotherapies for depression, because these have been examined most extensively. Furthermore, the perspective of different stakeholders will be presented: Patients, relatives, therapists, employers, health care providers and society at large each have their own perspectives on targets and outcomes. The perspective of patients should have more priority in research and standardization of measures across trials is much needed.

**Disclosure:**

No significant relationships.

